# Heat-Resistant *Aphanizomenon flos-aquae* (AFA) Extract (Klamin®) as a Functional Ingredient in Food Strategy for Prevention of Oxidative Stress

**DOI:** 10.1155/2019/9481390

**Published:** 2019-11-11

**Authors:** D. Nuzzo, M. Contardi, D. Kossyvaki, P. Picone, L. Cristaldi, G. Galizzi, G. Bosco, S. Scoglio, A. Athanassiou, M. Di Carlo

**Affiliations:** ^1^Istituto per la Ricerca e l'Innovazione Biomedica (IRIB), CNR, Via Ugo La Malfa 153, 90146 Palermo, Italy; ^2^Smart Materials, Istituto Italiano di Tecnologia (IIT), Via Morego 30, 16163 Genova, Italy; ^3^Dipartimento di Biopatologia e Biotecnologie Mediche (Di.Bi.Med.), Università di Palermo, Corso Tukory 211, 90134 Palermo, Italy; ^4^Le Farine dei Nostri Sacchi S.M.E., Via Ugo La Malfa 135, 90146 Palermo, Italy; ^5^Nutrigea-Nutritherapy Research Center, 61029 Urbino, Italy

## Abstract

Microalgae are generally considered an excellent source of vitamins, minerals, and bioactive molecules that make them suitable to be introduced in cosmetics, pharmaceuticals, and food industries. *Aphanizomenon flos-aquae* (AFA), an edible microalga, contains numerous biomolecules potentially able to prevent some pathologies including age-related disorders. With the aim to include an AFA extract (Klamin®) as a functional ingredient in baked products, we investigated if its bioactive molecules are destroyed or inactivated after standard cooking temperature. The AFA extract was exposed to heat stress (AFA-HS), and no significant decrease in pigment, polyphenol, and carotenoid content was detected by spectroscopic analysis. Thermal stability of AFA-HS extract was demonstrated by thermogravimetric analysis (TGA), and no change in the morphology of the granules of the powder was noticed by SEM microscopic observation. By Folin-Ciocalteu, ORAC, and ABTS assays, no change in the antioxidant activity and polyphenol contents was found after high-temperature exposition. When added in cell culture, solubilized AFA-HS lost neither its scavenging ability against ROS generation nor its protective role against Abeta, the main peptide involved in Alzheimer's disease. Prebiotic and antioxidant activities of AFA extract that are not lost after thermal stress were verified on *E. coli* bacteria. Finally, AFA-HS cookies, containing the extract as one of their ingredients, showed increased polyphenols. Here, we evaluate the possibility to use the AFA extract to produce functional food and prevent metabolic and age-related diseases.

## 1. Introduction

Microalgae comprise prokaryotic cyanobacteria and eukaryotic photoautotrophic protists, with significant diversity in their metabolism, cell structure, and habitat [1]. They are the oldest forms of life and have been the main biotic source of oxygen on early Earth [2]. These photosynthetic microorganisms exhibit commercial interest due to their ability to produce biomass from where bioactive compounds can be obtained. The last ones include pigments, vitamins, proteins, lipids, polyunsaturated fatty acids, and carbohydrates [3].

*Aphanizomenon flos-aquae* (AFA) is a cyanobacterial unicellular organism with remarkable nutritional properties that, unlike other commercial “microalgae,” spontaneously grows in Upper Klamath Lake (southern Oregon, USA). This lake is an ideal natural ecosystem for the growth of the AFA microalgae, especially during the period between late summer and early fall, while the cold is gradually starting. Sunny days favor the intense photosynthetic activity of AFA, which is achieved by its various pigments, including phycocyanins and phycoerythrins. Furthermore, the production of fatty acids, such as omega 3, is favored during the winter, while the lake freezes over due to low temperatures. Thanks to the volcanic origin of the lake, AFA contains a wide and complete variety of minerals [4, 5] and pigments such as carotene, beta-carotene, chlorophylls [5, 6], phycocyanins, phycoerythrins, and polyphenols [5, 7] with significant antioxidant [8], anti-inflammatory [5, 9], and antiproliferative [10] properties.

Numerous physiologically active biomolecules derived from algae have been investigated for their role in disease prevention and health and for their potential use as dietary supplements [11, 12]. For instance, Sabelli et al. [13] demonstrated the therapeutic benefits of phenylethylamine (PEA), an endogenous neuromodulator that is present in AFA algae and which, when deficient, can lead to certain forms of depression and affective disturbances [13, 14].

The nutritional supplement Klamin® is an AFA extract which contains concentrated quantity of phenylethylamine and other supporting molecules, and it has been proven to be effective in countering a wide variety of pathologies of the neurological system, including neurodegenerative diseases [15–17]. Oxidative stress, inflammation, and mitochondrial and neurological dysfunctions are some of the main causes of neurodegenerative pathologies, such as Alzheimer's disease (AD) [18–20]. In these years, the role of natural antioxidants as neuroprotective agents has been investigated in the molecular level, and their benefits have been widely demonstrated [18, 21–24]. These natural molecules can exert their antioxidant activity in two ways: by the improvement of the activity of antioxidant enzymes or by their scavenger capacity that can deactivate the reactive oxygen species (ROS). Therefore, the high levels of antioxidant molecules present in the AFA extract can make it a potential therapeutic agent, especially for those pathologies [19, 20].

In a precedent work, our group demonstrated that Klamin® shows scavenging properties against ROS generation and plays a role in mitochondrial protection [5]. In addition, it inhibits the aggregation process of beta-amyloid (Abeta), the main peptide involved in AD [5, 25, 26]. This peptide is a product of the sequential *γ*- and *β*-secretase proteolytic cleavage of the amyloid precursor protein (APP) [27]. Abeta has a high tendency to polymerize and form fibrils, aggregates, and the AD characteristic amyloid plaques, changing its conformation to a *β*-sheet structure [28]. However, several findings support the hypothesis that soluble oligomers, especially the ADDL aggregates, rather than plaques, are the most neurotoxic agents [29]. On this basis, synthetic or recombinant Abeta oligomers have been administered both in *in vitro* and *in vivo* model systems to mimic the AD pathology and study the possibility to inhibit its progression by using drugs or natural compounds [22, 23, 30–32].

The findings described above support the innovative and natural strategy that recommends the use of dietary supplements as an alternative form of prevention and treatment of several pathologies. So, the development of new medical formulations based on such supplements should be promoted [33–35]. Furthermore, dietary supplements can also be composed of extracts derived from different plants, and their health benefits against metabolic syndromes, arteriosclerosis, burns or chronic wounds, and neurodegenerative diseases have been proven [36–38]. Thus, dietary supplements could be recommended as an alternative form of prevention and treatment of different pathologies and could lead towards a more natural approach in the pharmaceutical industry.

One potential way in this new approach could be the development of functional food. In fact, numerous companies have shown interest in developing new strategies for the functionalization of food for the health and wellness market. Additionally, natural antioxidant compounds can also be used in the field of food packaging [39, 40] or as additives, preserving food from deterioration, rancidity, or discoloration. This allows extension of the shelf life without any adverse effects on the food's sensory or nutritional qualities. Moreover, these natural compounds can replace the currently used synthetic antioxidants such as butylated hydroxyanisole (BHA) or butylated hydroxytoluene (BHT) or propyl gallate (PG), that have also shown toxic effects, as food additives [41–43].

Finally, Sun et al. showed that anthocyanins extracted from Chinese purple sweet potato cultivar exert prebiotic-like activity, highlighting that bioactive molecules extracted from plants can be beneficial for the intestinal bacterial flora [44].

The goal of our research is to better analyze the activity of the bioactive molecules from the AFA extract and promote further investigation on its application in the food industry. More specifically, we analyzed a potential use of the AFA extract Klamin® as an addition to bread and/or biscuit dough, by investigating if its antioxidant and prebiotic activities would remain unaffected after its exposure to high temperatures.

## 2. Materials and Methods

### 2.1. AFA Extract Sample Preparation

The AFA extract Klamin® was kindly provided by Nutrigea Research s.r.l. (Republic of San Marino) [45]. The product was pulverized, according to Nuzzo et al. [5]. We called this sample AFA-Extract (AFA-E). For Heat-Stressed sample preparation, AFA-E powder was exposed at 220°C for 10 minutes, and this sample was called AFA-Heat Stressed (AFA-HS). For the experiments with cells and bacteria, a soluble fraction of the AFA extract was prepared. More specifically, 10 mg of AFA-E or AFA-HS powder was dissolved in 10 mL of PBS (pH = 7.4; 137 mM NaCl, 2.7 mM KCl, 8 mM Na_3_PO_4_). The solutions were sonicated (70% of the maximum power, twice for 30 seconds) and magnetically stirred for an hour. The insoluble fractions were removed by centrifugation at 14,000 rpm for 30 min at 4°C. The supernatants (soluble fractions) were collected, filtered by using a 0.45 *μ*m Sartorius filter, aliquoted (1 mL/vial), and stored at -20°C. These fractions are here named as AFA-soluble Extract (AFA-sE), and AFA-soluble Heat Stressed (AFA-sHS) (Table 1). For the experimental procedure, 10 mg from all the AFA samples was dissolved in 10 mL of PBS to achieve a concentration of 1 mg/mL.

### 2.2. Fluorescent Analysis of AFA Extracts

10 *μ*L of AFA-E and AFA-HS solutions was directly spotted onto nitrocellulose membrane strips and incubated at room temperature in the dark. After 20 minutes, their fluorescence intensity was measured by using a Typhoon FLA 9500 (GE Healthcare Life Sciences) fluorescence scanner at a resolution of 20 *μ*m. For fluorescence detection, a different wavelength was used, according to the pigment 635 nm (red laser) for phycocyanins, 532 nm (green laser) for phycoerythrins, and 473 nm (blue laser) for carotenoids. The fluorescence intensity was reported in arbitrary units (A.U.).

### 2.3. Chemical Characterization

The AFA-E and AFA-HS samples were chemically analyzed by Attenuated Total Reflection-Fourier Transform Infrared (ATR-FTIR) and UV-Vis spectroscopy. Infrared spectra were obtained with an ATR accessory (MIRacle ATR, PIKE Technologies) with a diamond crystal coupled to a Fourier-Transform Infrared (FTIR) spectrometer (Equinox 70 FT-IR, Bruker). All spectra were recorded in the range from 4000 to 600 cm^−1^ with a resolution of 4 cm^−1^, accumulating 128 scans. UV-Vis spectra of the powder of AFA-E and AFA-HS were taken by using a Cary 300 Scan UV-visible spectrophotometer.

### 2.4. Folin-Ciocalteu Colorimetric Assay

The phenolic content was calculated by using the Folin-Ciocalteu (F-C) colorimetric assay [5]. Aliquots (0.2 mL) of the AFA-E and AFA-HS extracts were made up to 5 mL with distilled water, and 0.5 mL of Folin-Ciocalteu reagent was added. After 3 min, 1 mL of Na_2_CO_3_ (20% *w*/*v*) was added to the reaction mixtures that were made up to 10 mL with distilled water. The samples were then stored for 2 hours at room temperature. The absorbance of the solutions was measured at 765 nm by using a spectrophotometer (Shimadzu) and was quantified by using a gallic acid standard curve.

### 2.5. Oxygen Radical Absorbance Capacity (ORAC) Assay

The ORAC assay was performed according to [46, 47], slightly modified. The reaction was carried out by using a 96-well plate: 160 *μ*L of 0.04 *μ*M fluorescein in 0.075 M Na-K phosphate buffer pH 7.0, 20 *μ*L of diluted phenolic extract, or 20 *μ*L of 100 *μ*M Trolox. The mixture was incubated for 10 min at 37°C in the dark. After this incubation, 20 *μ*L of 40 mM 2,2′-Azobis-(2-methylpropionamidine) dihydrochloride (AAPH) solution was added. The microplate was immediately placed in a microplate reader (Thermo Scientific Fluoroskan Ascent F2 Microplate), and the fluorescence was recorded (excitation and emission wavelengths at 485 and 527 nm, respectively) every 5 min for 60 min. The ORAC value refers to the net area under the curve of fluorescein decay in the presence of the Klamin® phenolic extract or Trolox, minus the blank area. The activity of the sample was expressed by *μ*mol of Trolox equivalents (TE)/g of AFA or AFA-Hs by using the following equation:

(1) $$\begin{array}{c} \text{ORAC}\left(\mu \text{mol TE}/\text{g}\right)=k*a*h*\left[\frac{\left(S_{\text{sample}}-S_{\text{blank}}\right)}{\left(S_{\text{Trolox}}-S_{\text{blank}}\right)}\right], \end{array}$$

where *k* is the final dilution of the water-soluble extract; *a* is the ratio between the volume (liters) of the water-soluble extract and the grams of AFA-E or AFA-HS; *h* is the final concentration of Trolox expressed as *μ*mol/L; and *S* is the area under the curve of fluorescein in the presence of sample, Trolox, or buffer solution. All the reaction mixtures were prepared in triplicates, and at least three independent assays were performed for each sample.

### 2.6. ABTS Free Radical Cation Scavenging Assay

The ABTS radical cation (ABTS^·+^) was generated by the reaction between 7 mM ABTS water solution with 2.45 mM potassium persulfate solution. The reaction took place in the dark at room temperature for 12-16 h. ABTS and potassium persulfate react stoichiometrically at a ratio of 1 : 0.5, resulting in the incomplete oxidation of the ABTS. The oxidation of the ABTS starts immediately, but the absorbance does not become maximal and stable until some hours are elapsed [48]. The ABTS^·+^ solution was then diluted with water to obtain a starting absorbance of 1.2 arbitrary units at 734 nm. Right after the dilution, 2 mg of AFA-E and AFA-HS was separately placed in polystyrene cuvettes containing 2 mL of the diluted ABTS^·+^ solution. The decrease of the absorbance was measured at 734 nm by using a Cary 300 Scan UV-visible spectrophotometer in the dark at room temperature for 24 h. All measurements were performed in triplicate. A sample with only 2 mL of ABTS^·+^ solution was used as a control, to ensure the stability of the solution. The radical scavenging activity (RSA) of AFA-E and AFA-HS was expressed as the inhibition percentage of the free radical of the samples and was calculated by using the following formula:

(2) $$\begin{array}{c} \text{Radical Scavenging Activity }\left(\%\right)=\left[\frac{{\left(A\right)}_{\text{control}}-{\left(A\right)}_{\text{sample}}\;}{{\left(A\right)}_{\text{control}}}\right]\times 100, \end{array}$$

where (*A*)_control_ stands for the absorbance of the control sample at a specific time point and (*A*)_sample_ for the absorbance of the sample at this time point. The results were presented in mean values with ±standard deviation (<1% for 24 h) [33, 49, 50].

### 2.7. Thermal Characterization

The thermal stability behavior of the AFA-E powder was investigated by a standard thermogravimetric analysis (TGA) method, using a TGA Q500 from TA Instruments. Measurements were performed with 3-5 mg samples in an aluminum pan under air atmosphere with a flow rate of 50 mL/min in a temperature range from 30 to 800°C and with a heating rate of 10°C/min. The weight loss and its first derivative were recorded simultaneously as a function of time/temperature. At the same operative conditions, an isothermal thermogravimetric characterization of the AFA-E sample was carried out, in order to simulate the cooking conditions. The AFA-E sample was exposed to a stable temperature of 220°C for 10 minutes, and the weight loss was evaluated. After 10 minutes, a thermal ramp from 220 to 800° with a heating rate of 10°C/min was performed to complete the thermal analysis.

### 2.8. Morphological Characterization

Morphology of the AFA-E and AFA-HS samples was analyzed by Scanning Electron Microscopy (SEM), using a variable pressure JOEL JSM-649LA microscope equipped with a tungsten thermionic electron source working in high vacuum mode, with an acceleration voltage of 5 kV. The specimens were coated with a 10 nm thick film of gold using a Cressington Sputter Coater-208 HR. The diameter of the granules of the powder was analyzed and determined by using the ImageJ software. Approximately 170 measurements were taken to obtain the diameter distribution of each algae sample.

### 2.9. Cell Cultures and Treatment

A549 human epithelial cells or LAN5 neuroblastoma cells were cultured with RPMI 1640 medium (Celbio srl, Milan, Italy) supplemented with 10% fetal bovine serum (Gibco-Invitrogen, Milan, Italy) and 1% antibiotics (50 mg mL^−1^ penicillin and 50 mg mL^−1^ streptomycin). Cells were maintained in a humidified 5% CO_2_ atmosphere at 37 ± 0.1°C. For toxicity assays, A549 cells were treated with 0.1, 0.5, and 1 *μ*g of AFA-sE and AFA-sHS for 24 hours or with H_2_O_2_ (50 *μ*M) pure or combined with AFA-sE and AFA-sHS at different concentrations (0.1, 0.5, and 1 *μ*g) for 24 hours. Untreated A549 cells were used as control. In the experiment that was carried out for the evaluation of the neurodegeneration effect, a recombinant Abeta peptide (60 *μ*M) was produced according to Carrotta et al. [51] and was administered to LAN5 cells under oligomeric form for 24 hours. Small oligomers (ranging between 8 and 67 kDa) were also prepared according to Carrotta et al. [51]. Briefly, after a preliminary treatment with trifluoroacetic acid (TFA), the powder of the recombinant Abeta was dissolved in 0.01 M Tris-HCl buffer, pH 7.2, and the solution was readily characterized by dynamic light scattering (DLS) at *T* = 15°C [26, 51].

### 2.10. Determination of Cell Viability

Cell viability was measured by the MTS assay (Promega Italia, S.r.l., Milan, Italy). MTS was used according to the manufacturer's instructions. After cell treatments, the incubation was carried out for 3 hours at 37°C, 5% CO_2_. The absorbance was measured at 490 nm on the Microplate Reader Wallac Victor 2 1420 Multilabel Counter (Perkin Elmer, Inc. Monza, Italy). Results were expressed as the percentage of the MTS reduction with the control samples as reference and presented as mean value ± standard deviation (SD).

### 2.11. Analysis of Reactive Oxygen Species (ROS) Generation

To assess ROS generation, treated A549 or LAN5 cells were placed in a 96-well microplate. Some of A549 were treated with H_2_O_2_ (50 *μ*M) alone or with the presence of AFA-sE (1 *μ*g) and AFA-sHS (1 *μ*g) or with AFA-sE (1 *μ*g) and AFA-sHS (1 *μ*g) as control for 24 hours. Then, dichlorofluorescein diacetate (DCFH-DA) (1 mM) was added to each sample, and then the samples were placed in the dark for 10 min at room temperature. After washing them with PBS, the cells were analyzed by a fluorescence microscope (Axio Scope 2 microscope; Zeiss, Oberkochen, Germany) and a fluorimeter Microplate Reader (GloMax, Promega) for fluorescence intensity detection.

### 2.12. Effect of AFA and AFA-HS on the Probiotic Bacteria Proliferation

The prebiotic activity of AFA-sE and AFA-sHS was tested on *Escherichia coli* (*E. coli*), a gram-negative bacterium, with a harmless serotype. One day before the test, a single colony was inoculated into Luria-Bertani (LB) liquid medium and incubated at 37°C overnight (o.n.). An aliquot (5 *μ*L) of the o.n. bacterial culture, approximately 10^9^ CFU/mL, was added to three test tubes containing fresh LB medium (5 mL). AFA-sE (1 *μ*g) or AFA-sHS (1 *μ*g) was added separately to the culture medium at time 0 min or 120 min. Untreated *E. coli* was used as control. For the bacteria oxidative stress experiment, an aliquot (5 *μ*L) of the o.n. bacterial culture was added to different test tubes containing fresh LB medium (5 mL) and H_2_O_2_ at different concentrations (1, 2, 3, and 4 mM) was added when the bacteria were in the exponential phase of growth (*data not shown*). For the inhibition of the oxidative stress experiment, bacteria were incubated with H_2_O_2_ (1.5 mM) in three different test tubes, and when the bacteria were in the exponential phase of growth, AFA-sE (1 *μ*g) or AFA-sHS (1 *μ*g) was separately added. *E. coli* treated with H_2_O_2_ or AFA-sE or AFA-sHS was used as control. In all of the experiments, the exponential growth was determined by reading the absorbance value at 600 nm (OD_600_) at a spectrophotometer with 30 min intervals.

### 2.13. Effect of AFA and AFA-HS on the Bacteria Reactive Oxygen Species (ROS) Generation

An aliquot of *E. coli* o.n. culture solution, approximately 10^9^ CFU/mL, was diluted (1 : 10^5^) and 5 *μ*L was placed in a 96-well optical bottom white microplate. H_2_O_2_ (1.5 mM) with AFA-sE (1 *μ*g) or AFA-sHS (1 *μ*g) was added to the wells. *E. coli* treated with H_2_O_2_ or AFA-sE or AFA-sHS was used as control. Then, the samples were incubated with 1 mM DCFH-DA for 2 and 4 hours at room temperature. Afterward, the *E. coli* samples were analyzed by using the Microplate Reader (GloMax, Promega) for fluorescence detection.

### 2.14. Functional Food Design

The cookie dough was prepared according to “*Le Farine dei Nostri Sacchi* s.r.l.” (Palermo, Italy) by using classical ingredients with the addition of Klamin® or AFA-E (Table 2) and baking at 220°C for 10 minutes. Each cookie (1.5 g) contains 0.1 g of AFA-E. For the F-C assay, 2 g of biscuit dough raw or cooked was dissolved in 15 mL of water and, after vortexing, centrifuged for 20 min at 1500g. Then, 0.5 mL of the F-C reagent was added to 2 mL of the filtered supernatant and the analysis was performed as described above.

### 2.15. Statistical Analysis

The significance of the differences in the mean values of multiple groups was evaluated by using one-way analysis of variance (ANOVA) followed by Bonferroni's post hoc test. Differences were considered significant when the *p* value was ≤0.05.

## 3. Results

### 3.1. Heat Stress Does Not Affect AFA Extract Content and Antioxidant Activity

**Figure 1:**
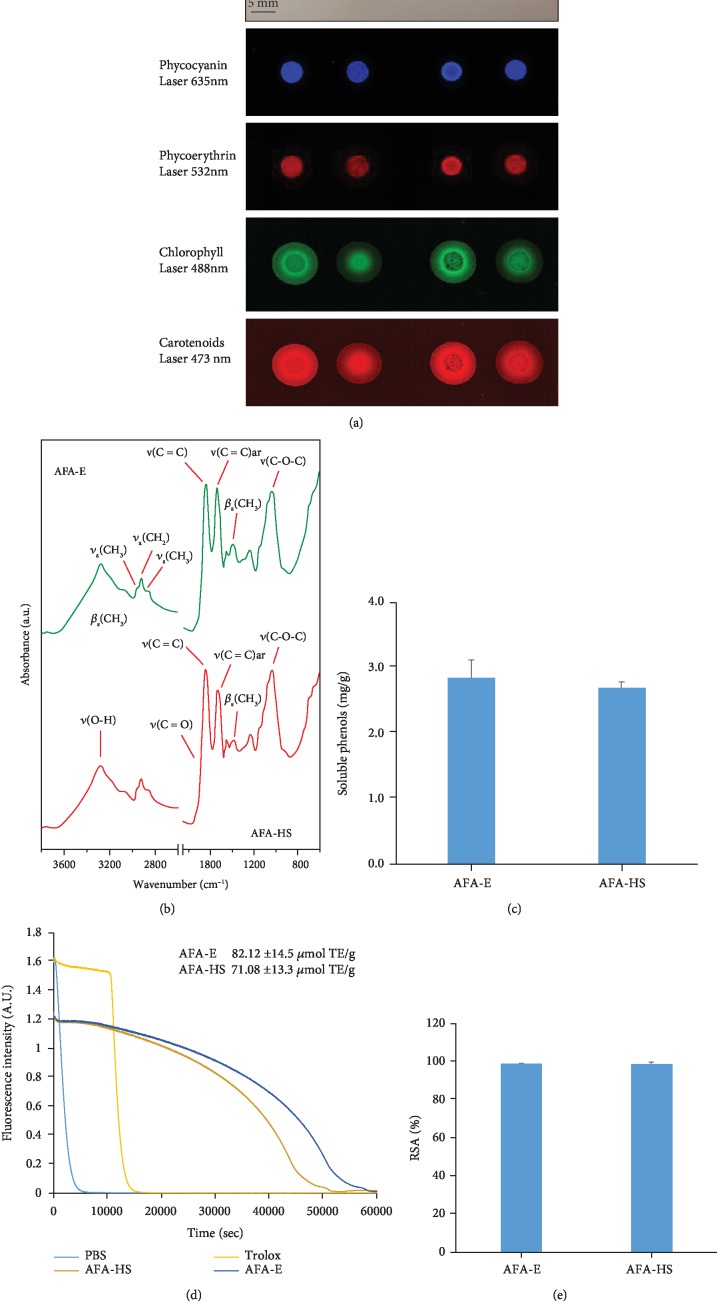
Fluorescence measures and absorbance quantification of AFA extracts under no-thermic (AFA-E) and thermic stress (AFA-HS). (a) Fluorescence intensity at 635 nm (phycocyanins), 532 nm (phycoerythrins), 488 nm (chlorophyll), and 473 nm (carotenoids). (b) Chemical analysis, ATR-FTIR spectra of the AFA-E (top) and AFA-HS (bottom) samples. (c) Polyphenol contents of the AFA-E and AFA-HS assayed by Folin-Ciocalteu. (d) Antioxidant capacity of the AFA-E and AFA-HS assayed by ORAC reducing capacity. (e) ABTS assays, comparison between the radical scavenging activity percentages of the AFA-E (left) and the AFA-HS (right) after 24 h.

To evaluate if the pigment contents of AFA extracts are maintained under heath stress, we performed an analysis based on their spectroscopic properties and chromophore content [52]. By using appropriate excitation and emission filters, no significant difference in fluorescence intensity and absorption spectra was detected between unheated (AFA-E) and heated (AFA-HS) phycoerythrin and phycocyanin samples. However, light differences were observed for chlorophyll and carotenoids (Figure 1(a)), indicating a minor stability with respect to the other pigments. The ring around the AFA spots (Figure 1(a)) was due to the diffusion of chlorophyll and carotenoids [53].

Infrared spectra of the AFA-E and AFA-HS samples are reported in Figure 1(b). AFA-E is a mix of compounds, so various bands can be associated with different chemical structures, such as polyphenols, pigments, and carotenoids. The two spectra were mainly characterized by the following bands: O-H stretching mode at 3281 cm^−1^, asymmetric CH_3_ stretching mode at 2957 cm^−1^, asymmetric and symmetric CH_2_ stretching mode at 2926 and 2874 cm^−1^, respectively, C=O stretching mode at 1685 cm^−1^, C=C stretching mode at 1643 cm^−1^, aromatic C=C stretching mode at 1533 cm^−1^, symmetric CH_3_ binding mode at 1387 cm^−1^, and C-O-C stretching mode at 1034 cm^−1^. Differences between AFA-E and AFA-HS spectra in the intensity of the peak at 1533 cm^−1^ and at 1387 cm^−1^ were observed. The first one is the stretching of aromatic C=C conjugated with aliphatic C=C, and it is typical of phenolic compounds [54]. Instead, the peak at 1387 cm^−1^ can be assigned to the CH_3_ present in the carotenoid structure [55]. Therefore, due to the heating treatment, a decrease in the quantity of these compounds present in the AFA-E powder was observed. After that, the maintenance of polyphenols and bioactive activity after thermic stress was analyzed by Folin, ORAC, and ABTS assays. The values were expressed as mg of gallic acid equivalents per g of extract for the Folin-Ciocalteu assay (Figure 1(c)) and as *μ*mol of Trolox equivalents (TE) per g of extract for ORAC assays (Figure 1(d)), and no significant differences were detected between AFA-E and AFA-HS samples. Finally, by the ABTS radical cation assay, we measured the reduction of the radical cation as the inhibition percentage of absorbance at 734 nm. The comparison between the antioxidant activity of the AFA-E and AFA-HS is presented in Figure 1(e). More specifically, the reaction of our samples with ABTS^·+^ was completed within 24 h, reaching a percentage of 99.1% and 98.6% for AFA-E and AFA-HS, respectively (Figure 1(e)). These results clearly demonstrate that both AFA-E and AFA-HS were able to inhibit successfully the free radicals from the ABTS^+^ solution.

### 3.2. Thermal Stability and Morphological Analysis

**Figure 2:**
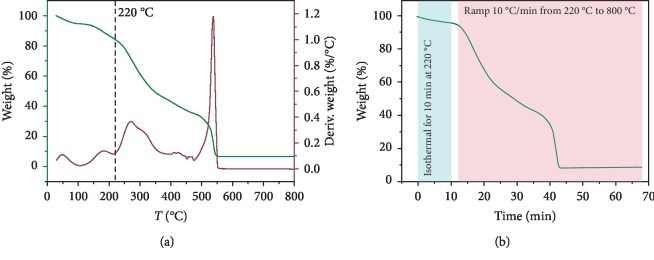
Thermal stability analysis. (a) TGA curve of AFA-E expressed as weight percentage (left side) and its first derivate (right side) in the range of temperature between 30 and 800°C. (b) Isothermal thermogravimetric analysis of AFA-E sample at 220°C (blue area) followed by a nonisothermal thermogravimetric analysis (pink area) from 220°C to 800°C at the rate of 10°C/min as a function of the time.

The thermal properties of the AFA-E sample were evaluated by TGA, and the main results are reported in Figure 2.  Figure 2(a) shows the thermogravimetric analysis of AFA-E expressed as weight loss percentage (left side) and its first derivate (right side). The powder showed a weight loss of ≈16% when it reached a temperature of 220°C (dash line in Figure 2(a)). Instead, it is decomposed at 550°C, and at the end of the thermogravimetric measurement, a final residual of ≈6.5% was observed.

**Figure 3:**
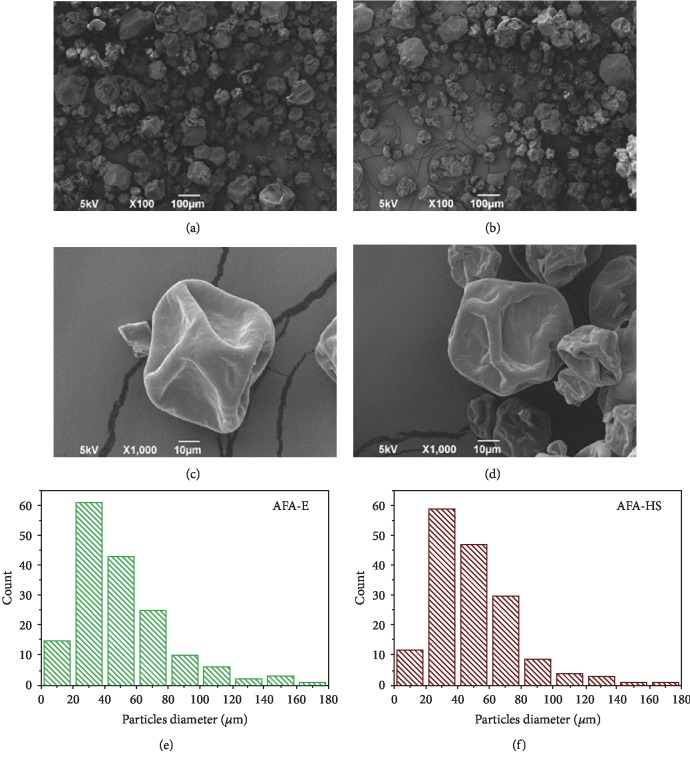
Morphological analysis. (a, c) SEM images of the AFA-E powder at different magnifications. (b, d) SEM images of the AFA-HS powder at different magnifications. (e, f) Particle diameter distribution for the AFA-E and AFA-HS samples, respectively.

In Figure 2(b), an isothermal analysis for the AFA-E powder is reported. In order to evaluate the potential weight loss in cooking condition, we maintained the temperature of 220°C for 10 min. After 10 min at the cooking temperature, the powder lost only 4% of its weight, demonstrating its potential compatibility with preparation procedure of bakery products. Afterward, the temperature was increased from 220 to 800°C, and the powder showed the same trend observed in Figure 2(a) with a total decomposition achieved at 550°C. Furthermore, the morphology of the AFA-E and AFA-HS samples was analyzed by SEM. Figures 3(a) and 3(c) show two top-view images at different magnifications of a characteristic AFA-E sample, whereas Figures 3(b) and 3(d) report the top-view images of the AFA-HS sample. No differences in shape were noticed between the sample before and after the thermal treatment, and the “deflated ball” shape was maintained. In Figures 3(e) and 3(f), the diameter size distribution of the particles is presented for the AFA-E and AFA-HS samples, respectively. In both cases, a main distribution between 20 and 80 *μ*m was observed.

### 3.3. AFA-sE and AFA-sHS Recover H_2_O_2_-Induced Cytotoxic Effect

**Figure 4:**
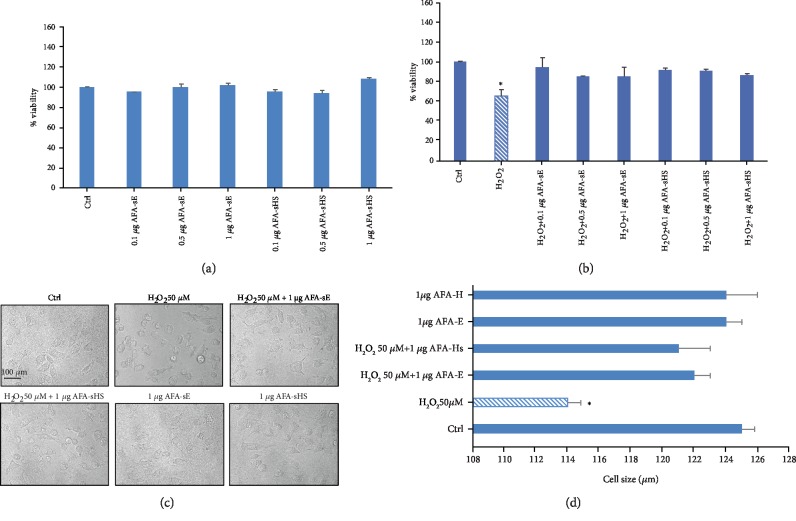
Effect of AFA-sE and AFA-sHS on A549 cells. (a) MTS cell viability assay of A4509 cells alone (Ctrl) or incubated with different AFA-sE and AFA-sHS concentrations. (b) MTS cell viability assay without (Ctrl) or after treatment of A549 cells with H_2_O_2_ alone or in combination with different AFA-sE and AFA-sHS concentrations. (c) Representative morphological images of A549 untreated cells (Ctrl) or treated with AFA-sE and AFA-sHS or with H_2_O_2_ alone or in combination with AFA extracts. (d) Histogram of A549 untreated cells (Ctrl) or treated with AFA and AFA-HS cell body size. Bar: 100 *μ*m.

In order to understand if AFA-E is able to release toxic molecules after heat treatment, different concentrations of untreated (control) and high temperature-treated AFA extracts were added to A549 cells, and after incubation for 24 hours, an MTS assay was performed.  Figure 4(a) shows that no toxicity was detected at all the concentrations, compared with the control. Furthermore, to assess if unheated or heated AFA extracts can inhibit H_2_O_2_-induced toxicity, A549 cells were incubated with hydrogen peroxide in combination with different concentrations of AFA-sE and AFA-sHS. As shown by the MTS assay, all of the used AFA extracts are able to inhibit the H_2_O_2_-induced cell toxicity, and no differences were observed for the heated samples (Figure 4(b)). All these results were confirmed by the morphological observation (Figure 4(c)) and analysis of the cell body size in which a recovery of the altered cell shape due to H_2_O_2_ treatment was detected (Figure 4(d)).

### 3.4. AFA-sE and AFA-sHS Inhibit ROS Generation

**Figure 5:**
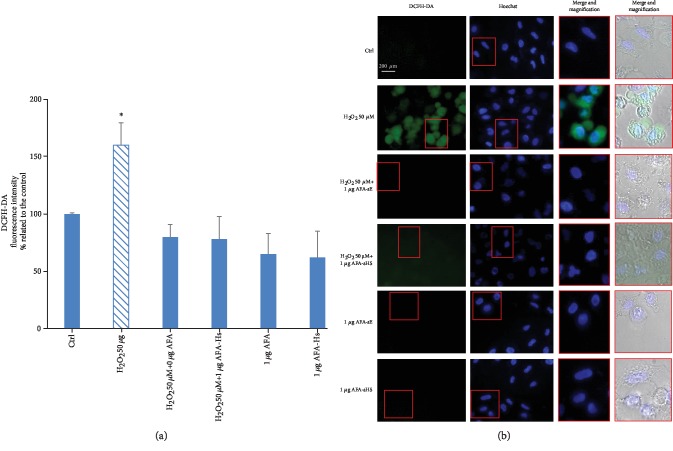
AFA extracts protect A549 cells from oxidative insult. (a) Fluorescence intensity of A549 cells alone (Ctrl) or treated with H_2_O_2_ or AFA-sE or AFA-sHS alone or cotreated with H_2_O_2_ and AFA-sE or AFA-sHS measured by the DCFH-DA assay. (b) Fluorescence microscopy images of untreated cells (Ctrl) and cells treated with H_2_O_2_ or cotreated with H_2_O_2_ and the AFA extracts.

A clear-cut result about the maintenance of the antioxidant activity of the AFA extract after thermal treatment was evaluated by treating A549 cells with H_2_O_2_ alone or in combination with unheated and heated AFA extracts and by using the DCFH-DA assay. By fluorometric analysis, we detected that the presence of both of AFA-sE and AFA-sHS decreases H_2_O_2_-induced ROS generation (Figure 5(a)). Furthermore, these data were confirmed by fluorescence microscope inspection. Indeed, cells treated with H_2_O_2_ showed green fluorescence due to ROS generation, while cells treated with AFA extracts or H_2_O_2_ AFA-treated extracts did not show any fluorescence (Figure 5(b)). The result suggests that the components of AFA extract, such as carotenoids, phycoerythrins, phycocyanins, and polyphenols, preserve a significant role as antioxidant agents even if they are heat stressed.

### 3.5. Neuroprotective Effect of AFA-sE and AFA-sHS

**Figure 6:**
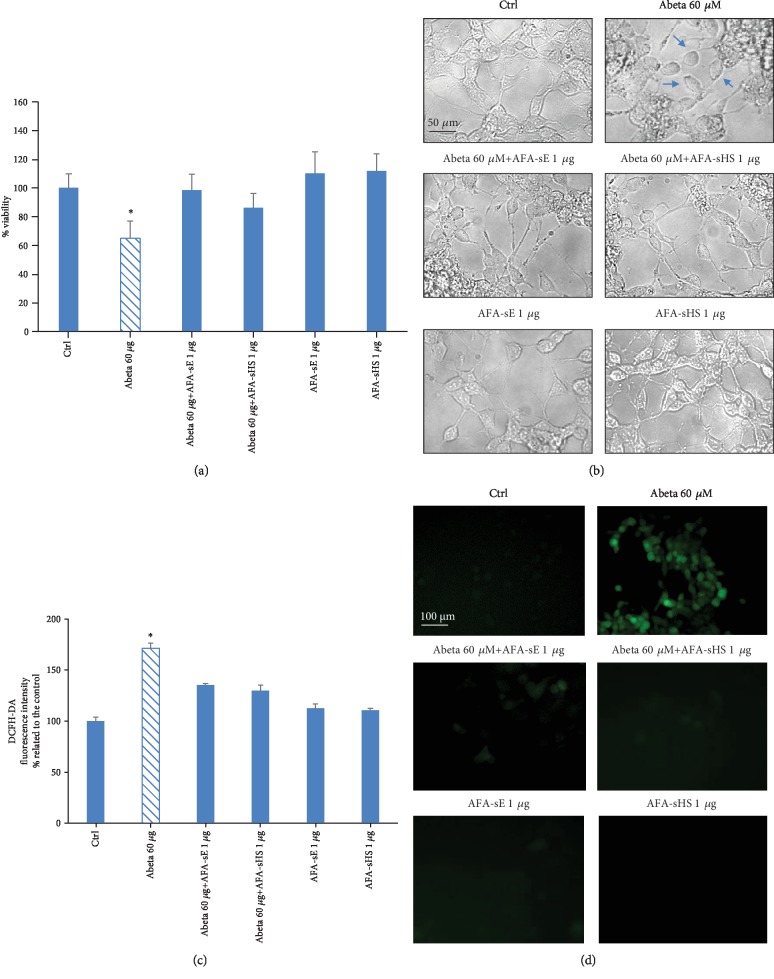
AFA-sE and AFA-sHS protect against Abeta-induced toxicity. (a) MTS of untreated LAN5 cells (Ctrl) or cells treated with the AFA extract, with Abeta alone, or with AFA-sE or AFA-sHS. (b) Morphological representative images of samples indicated in (a). (c) DCFH-DA assay of untreated LAN5 cells (Ctrl) or cells treated with Abeta oligomers alone or with AFA-sE or AFA-sHS. (d) Fluorescence representative images of samples indicated in (c). Bar: 50 *μ*m.

Since a neuroprotective effect was demonstrated for Klamin® supplement [5], we tested if this property is maintained after thermic stress. LAN 5 cells were treated with Abeta oligomers alone or with AFA-sE and AFA-sHS extracts and submitted to the MTS assay. The Abeta-induced toxicity was inhibited by the coadministration of the AFA extracts (Figure 6(a)). Observation of cell morphology confirmed the viability assay results (Figure 6(b)). The antioxidant capacity of both AFA extracts against the Abeta oligomer-induced oxidative stress was evaluated by the DCFH-DA assay. Fluorescence analysis indicated that cells treated with Abeta alone exhibit high levels of ROS generation, whereas cotreatment of Abeta oligomers and AFA extracts did not produce any fluorescent signal (Figure 6(c)). The same samples were observed at fluorescent microscopy (Figure 6(d)).

### 3.6. Prebiotic and Antioxidant Effect of AFA-sE and AFA-sHS on Bacteria

**Figure 7:**
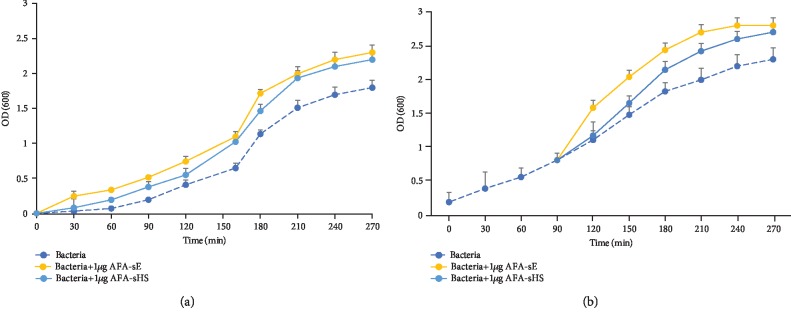
AFA and AFA-HS have prebiotic effect. *E. coli* growth curve with the presence of AFA-sE and AFA-sHS added at the beginning (a) or at the exponential phase of growth (b).

Prebiotics beneficially affect the intestinal microbiota, stimulating the growth or activity of helpful bacteria and altering their composition [55, 56]. The prebiotic effect of AFA-sE and AFA-sHS on *Escherichia coli* was evaluated by following the growth curve. AFA-sE and AFA-sHS were added to *E. coli* culture at time 0 (Figure 7(a)) or when the bacteria were in the exponential phase of growth (Figure 7(b)). In both cases and samples, an increase in the growth of bacteria was observed with respect to the control, indicating that AFA extracts induce a prebiotic proliferation activity of bacteria that is not affected by the heat stress.

**Figure 8:**
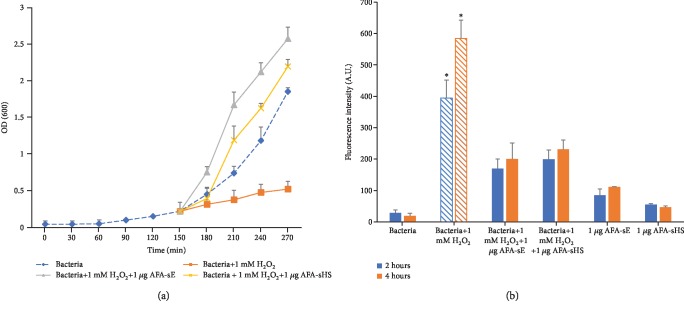
AFA extracts protect *E. coli* by oxidative stress. (a) Growth curve of *E. coli* alone (bacteria) or treated with H_2_O_2_ alone or with H_2_O_2_ in combination with AFA-sE and AFA-sHS. (b) Oxidation kinetics of *E. coli* alone (bacteria) or in the presence of H_2_O_2_ or H_2_O_2_ in combination with AFA-sE and AFA-sHS measured by the DCFH-DA assay after 2 and 4 hours of incubation.

The bacterial cell envelope is mainly exposed to the oxidizing molecules generated by the extracellular environment or host cells, and although bacteria possess defense mechanisms against oxidative stress, they sometimes could be inadequate. To evaluate whether AFA extracts can inhibit H_2_O_2_-induced toxicity or not, we exposed bacteria in the exponential phase of growth to peroxide alone or in combination with AFA-sE or AFA-sHS and a recovery of toxicity was observed (Figure 8(a)). Furthermore, AFA-sE and AFA-sHS antioxidant ability was analyzed after 2 and 4 hours by using the DCFH-DA assay. The presence of AFA-sE and AFA-sHS decreases H_2_O_2_-induced ROS generation in a time-dependent manner (Figure 8(b)). No significant differences in the antioxidant activity were observed between the unheated and heated samples.

### 3.7. Functional Cookies' Preparation

**Figure 9:**
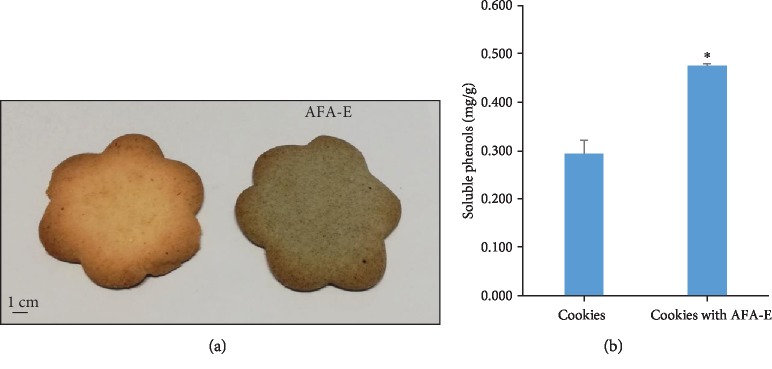
AFA extract is a versatile ingredient for functional food. (a) Biscuit dough without or with AFA-E after cooking. (b) Polyphenol content in cookies without or with AFA.

The basic cookie dough was prepared according to a classic recipe with the addition of AFA-E and baked at 220°C for 10 minutes (Figure 9(a)). Furthermore, the presence of polyphenols before and after the baking of the biscuits was analyzed by the F-C assay (Figure 9(b)). AFA addition improved the polyphenol contents, and no significant differences were detected after cooking, indicating that AFA is a useful functional compound.

## 4. Discussion

On the basis of current knowledge, food may not only be considered as essential for the survival of humans but also as a delight for the palate, promoter of well-being, and help in reducing the risk of diseases. This might be particularly important considering the growing cost of health care, the increase in life expectancy, and the continuous request for a better quality of life. Functional foods are conventional foods with additional functions often related to health promotion or disease prevention. These products are designed to offer a reduction in the risk of developing some diseases and a control of metabolic parameters, such as cholesterol levels, blood sugar concentration, oxidative stress, and inflammation [57–59]. Thus, in a view of a varied and balanced human diet, AFA extract could be an excellent source of vegetable nutrients and bioactive components.

High temperature slightly affects only chlorophyll and carotenoids, but as demonstrated by classical assays, the AFA-E antioxidant properties were preserved, indicating that the undamaged molecules exert a synergic compensatory effect.

As suggested by the isothermal thermogravimetric analysis, the AFA extract can resist the thermal treatment, showing a reduction of just 4% of its initial weight when exposed to a stable temperature of 220°C. Moreover, a slight reduction of the aromatic and carotenoid part of the AFA was observed in the ATR-FTIR spectra. Finally, no morphological changes were noticed in the powder structure. Thus, AFA extract can be a suitable ingredient, which meets the functional food requirements. Taking dietary antioxidants could indeed be useful in cases in which endogenous antioxidants are not sufficient to counteract the oxidative processes that can take place in the human body in case of some pathologies or during the natural aging process [60]. In agreement with our results, studies in which *Vicia narbonensis L*. (Narbon bean) extract was used to produce gluten-free functional crackers have demonstrated that the antioxidant capacity of its compounds is not only unaffected and but also possibly increased during baking [61]. The presence and thermic-stress resistance of AFA bioactive compounds make them suitable as food ingredients and as preservatives against deterioration due to oxidation, improving the stability and extending the shelf life of food products [62].

Neurodegeneration is characterized by oxidative stress process in specific areas of the brain, and antioxidants may have a positive effect in this kind of pathologies. Daily consumption of food containing bioactive molecules with antioxidant properties could be an approach for the prevention and treatment against neurodegenerative diseases. The beneficial effect on neurons in which toxicity was induced by Abeta oligomers was also maintained after thermal stress. This data suggests that the AFA extract could play a significant neuroprotective role if consumed as an ingredient of functional food. However, its protective role could also be exerted by direct interaction on Abeta aggregation and amyloid plaque formation [5].

On the basis of the reported results in which the biological activity of the AFA extract is maintained during baking, we cannot exclude that the same could also be used to produce other kind of functional foods such as pasta, one of the main ingredients of the Mediterranean Diet (MedDiet) [63] that are cooked at 100°C. In a recent study, pasta enriched with 3% of *Opuntia ficus-indica* (OFI) extract, a plant known for its antioxidant and anti-inflammatory properties, was given to volunteers and beneficial effects were observed [64]. Similarly, pasta with beta-glucans as functional food has been investigated in a pilot study, demonstrating that its consumption could help in the prevention of age-related metabolic disorders [65].

Many studies have proven that microbiota plays a relevant role in the maintenance of the proper function of the gastrointestinal system, and several systemic disorders lead to its imbalance. Obesity and other metabolic-related disorders can cause gut microbiota dysbiosis. Fortunately, numerous bioactive compounds from the diet have a significant influence on its composition and could be useful tools against these pathologies [66]. It has been reported that milk fat globule membrane (MFGM) supplementation is able to modulate gut microbiota composition, demonstrated by the fact that a beneficial effect was observed in mice, after following a diet rich in fats. In addition, the possibility to use MFGM as a potential ingredient in functional food for dietary strategies against metabolic disorders has been suggested [67].

Further, antioxidant molecules such as resveratrol improved the mouse gut microbiota dysbiosis induced by high-fat diet by increasing the *Bacteroides/Firmicutes* ratio [68]. In agreement with these findings, we observed that the AFA extract has a prebiotic effect and an antioxidant activity that are not lost after thermal stress, indicating that it could regulate the gut microbiota composition. AFA extract could also exert its protective role by modulating the gut microbiota profile. Furthermore, since metabolic disorders are a risk factor for neurodegeneration [69], we cannot exclude the possibility that AFA extract could also prevent age-related disorders via gut microbiota modulation.

AFA biscuits showed increased polyphenol content with respect to the traditional recipe without AFA, indicating that their antioxidant properties were not only maintained but also enhanced after baking the cookies at a high temperature. Our preliminary results let us speculate that the AFA cookies could be considered as a functional food for the prevention and management of pathologies in which oxidative stress is a risk factor, including metabolic and age-related disorders. Additional studies on healthy volunteers to whom cookies are administered will help us to validate the effect of AFA cookies on human health.

## Figures and Tables

**Table 1:** 

Abbreviations	
AFA-E	Powder of AFA extract
AFA-HS	Heat-stressed powder of AFA
AFA-sE	Hydrosoluble extract of AFA-E
AFA-sHS	Hydrosoluble extract of AFA-HS

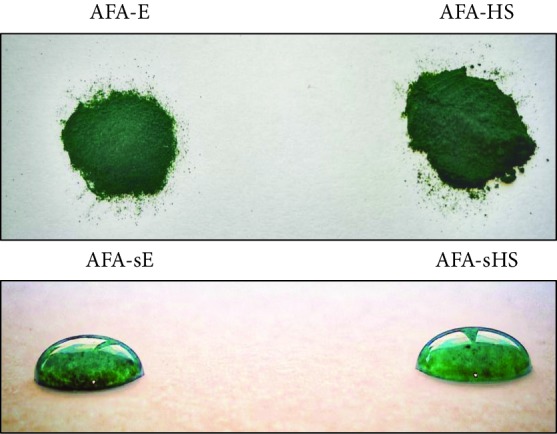	

**Table 2:** 

Ingredients	
500 g	Mix of flour
126 g	Margarine
186 g	Sugar
3 g	Yeast
93 g	Eggs
6 g	AFA-E
19.5 mL	Water

## Data Availability

The data used to support the findings of this study are available from the corresponding author upon request.
